# The roles of integrin β1 in phenotypic maintenance and dedifferentiation in chondroid cells differentiated from human adipose-derived stem cells

**DOI:** 10.1186/1556-276X-8-136

**Published:** 2013-03-24

**Authors:** Simin Luo, Qiping Shi, Zhengang Zha, Ping Yao, Hongsheng Lin, Ning Liu, Hao Wu, Jiye Cai, Shangyun Sun

**Affiliations:** 1 The First Affiliated Hospital, Jinan University, Guangzhou, 510632, China; 2 Institute of Orthopaedic Disease Research, Jinan University, Guangzhou, 510632, China; 3 School of Medicine, Jinan University, Guangzhou, 510632, China; 4 Department of Chemistry and Institute for Nano-Chemistry, Jinan University, Guangzhou, 510632, China

**Keywords:** Integrin β1, Adipose-derived stem cells, Dedifferentiation, Atomic force microscope

## Abstract

**Objective:**

The aim of this study is to probe the intrinsic mechanism of chondroid cell dedifferentiation in order to provide a feasible solution for this in cell culture.

**Methods:**

Morphological and biomechanical properties of cells undergoing chondrogenic differentiation from human adipose-derived stem cells (ADSCs) were measured at the nanometer scale using atomic force microscopy and laser confocal scanning microscopy. Gene expression was determined by real-time quantitative polymerase chain reaction.

**Results:**

The expression of *COL II, SOX9*, and *Aggrecan* mRNA began to increase gradually at the beginning of differentiation and reach a peak similar to that of normal chondrocytes on the 12th day, then dropped to the level of the 6th day at 18th day. Cell topography and mechanics trended resembled those of the genes’ expression. Integrin β1 was expressed in ADSCs and rapidly upregulated during differentiation but downregulated after reaching maturity.

**Conclusions:**

The amount and distribution of integrin β1 may play a critical role in mediating both chondroid cell maturity and dedifferentiation. Integrin β1 is a possible new marker and target for phenotypic maintenance in chondroid cells.

## Background

Adipose-derived stem cells (ADSCs) are multipotent cells that can differentiate into cells of multiple tissue lineages, such as osteocytes, chondrocytes, adipocytes, or neuronal cells. Recent research has indicated that ADSCs can differentiate into chondrocytes in vitro, but chondroid cells ultimately lose their phenotype, or dedifferentiate, in long-term culture through a poorly understood mechanism [1,2]. Over the past several years, in order to maintain or reinstate differentiation of chondrocytes, cultures were supplemented with exogenous cytokines, such as PTHrP [3], exogenous bone morphogenetic protein (BMP)-2 [4], triiodothyronine (T3) [5], fibroblast growth factor 18 [6], and electroporation-mediated transfer of *SOX* trio genes (*SOX-5*, *SOX-6*, and *SOX9*) to mesenchymal cells [7]. Additional methods to prevent dedifferentiation include changing culture systems to those similar to microcarriers [8], high-density micromass culture [9], three-dimensional (3D) cultures in hydrogels [10], in pellet culture using centrifuge tubes [11], and 3D dynamic culture using 3D-stirred suspension bioreactor (spinner-flask) culture system [12].

The cell membrane plays an important role in cell physiology and in regulating processes such as material transport, energy conversion, signal transduction, cell survival, apoptosis, and differentiation [13-15]; so alteration of the cell surface ultrastructure can directly influence cellular function [16]. Despite its importance, there are still many unanswered questions about the role of the cell membrane in differentiation: whether there are changes or defects on cellular membrane later in differentiation, whether these defects during late stage differentiation cause dedifferentiation by disturbing cellular homeostasis, and whether the biophysical properties in plasma membrane could be manipulated to maintain differentiation or redifferentiate the cell.

Atomic force microscopy (AFM) has recently emerged as an implement to image the cell membrane and detect mechanical properties at nanometer scale [17]. We are the first to use AFM to observe the change in morphological and biomechanical properties between chondroid cells and normal chondrocytes, leading to the detection of plasma membrane proteins at the molecular scale. We also used flow cytometry and laser confocal scanning microscopy (LCSM) to analyze integrin β1 expression during chondrogenic differentiation of ADSCs. We used these techniques to probe the intrinsic mechanism of chondroid cell dedifferentiation in order to provide a feasible solution for this in cell culture.

## Methods

### ADSCs isolation, culture, and identification

Subcutaneous adipose tissue was resected from seven patients (mean age, 26 years; range, 12 ~ 32 years) undergoing inguinal herniorrhaphy. Research ethics board approval for this study was obtained from Jinan University. Isolation and identification of ADSCs was performed as described previously [18] with modifications. Cells were cultured in DMEM/F12 (Gibco, Invitrogen, Carlsbad, CA, USA) supplemented with 10% fetal bovine serum (FBS, Gibco, USA) and 1% antibiotic (100 U/ml penicillin and 0.1 mg/ml streptomycin, Sigma-Aldrich Corporation, St. Louis, MO, USA) in an incubator (5% CO2, 37°C). The medium was refreshed every 3 days, and cells were split 1:3 after reaching 90% confluence.

### Chondrogenic differentiation

ADSCs (passage 3) were seeded at a high-cell density (2 × 10^5^/10 ml), then the medium was changed to DMEM/F12 supplemented with chondrogenic medium: 1% FBS, 6.25 μg/ml insulin + ITS (Sigma, USA), 10 ng/ml TGF-β1 (Peprotech, Rocky Hill, NJ, USA), 10 to 7 M dexamethasone (Sigma, USA), 50 μg/ml ascorbic acid (Sigma, USA), 100 U/ml penicillin, and 0.1 mg/ml streptomycin as previously described [18]. Twenty-one days after induction, lipid accumulations in adipocytes were visualized by staining with oil red-O as follows: cells were fixed in 10% formalin for 1 h and stained for lipid with 0.3% oil red-O for 15 min. After rinsing three times with double distilled H_2_O, the red-staining cells in six random areas of 1 mm^2^ were counted in each well and presented as an average ± standard deviation for 3 to 6 replicate wells.

### Chondrocytes isolation and culture

Cartilage was obtained from six patients (mean age, 58 years; range, 40 ~ 78 years) undergoing total hip replacement at the First Affiliated Hospital of Jinan University, with femoral neck fracture. Chondrocytes were isolated and collected according to the procedure proposed by Malicev et al. [19], with slight modifications. Culture medium contains DMEM/F12 supplement with 10% FBS.

### Primer design

The primers for amplification of *Aggrecan*, *COLII*, *SOX9*, and *COLI* were designed using Primer Express 5.0 software using default parameters according to the published sequences in Gen-Bank. Glyceraldehyde-3-phosphate dehydrogenase (GAPDH) was used as a positive control. The primer sequences are listed in Table 1. All primers were obtained from Invitrogen.

**Table 1:** Sequences of primers for real-time PCR

**Primer name**	**Forward primer (5**^**′**^**-3**^**′**^**)**	**Reverse primer (5**^**′**^**-3**^**′**^**)**	**Product size (bp)**
*Aggrecan*	5^**′**^-CTGCCCCAGAAGTGAGTGGAG-3^**′**^	5^**′**^-TGGTGCTGATGACAACGCCC-3^**′**^	159
*COL II*	5^**′**^-CACCTGCAGAGACCTGAAA-3^**′**^	5^**′**^-CAAGTCTCGCCAGTCTCCAT-3^**′**^	126
*Sox-9*	5^**′**^-AACGCCATCTTCAAGGCG-3^**′**^	5^**′**^-CTCTCGCTTCAGGTCAGCCTT-3^**′**^	165
*COL I*	5^**′**^-CCTGGATGCCATCAAAGTCT-3^**′**^	5^**′**^-ACTGCAACTGGAATCCATCG-3^**′**^	150
GAPDH	5^**′**^-CCACCATGGAGAAGGCTG-3^**′**^	5^**′**^-GGTGCTAAGCAGTTGGTCCT-3^**′**^	170

### RNA isolation and real-time-polymerase chain reaction analysis

Total RNA was extracted using Trizol (Invitrogen, USA) protocol. Two micrograms of total RNA was used for reverse transcription reaction with the RevertAid First Strand cDNA synthesis kit (Fermentas, Thermo Fisher Scientific Waltham, MA, USA) and random oligo(dT) primer (Fermentas), according to the manufacturer’s instructions. The cell was collected at different time points after differentiation (0, 3, 6, 9, 12, 15, 18, and 21 days), and expression of *Aggrecan*, *COLII*, *SOX9*, *COLI*, and *GAPDH* genes in the regenerated fragments was measured by real-time polymerase chain reaction (RT-PCR). Samples were set up in duplicate with the Power SYBR® Green and analyzed with the ABI 7500 Real-Time PCR System (Applied Biosystems, Life Technologies Corp., Carlsbad, CA, USA). RT-PCR was performed using PCR Taq core kit (Takara Bio Inc., Dalian, China).

### Single cell atomic force microscopy measurement

The cells were fixed with 2.5% glutaraldehyde for 15 min, then washed three times with distilled water. Morphology and mechanical response of cells were obtained by AFM (Autoprobe CP Research, Veeco, Plainview, NY, USA) imaging under contact mode. All data were analyzed with the instrument-equipped software IP2.1. silicon nitride tips (UL20B, Park Scientific Instruments, Suwon, South Korea) were used in all AFM measurements. In each group, single-cell imaging was repeated for six cells, and each cell was scanned three times. The nominal tip curvature radius was less than 10 nm; a spring constant of silicon cantilevers was 0.01 N/m; a resonance frequency was 285 kHz; the loading force was adjusted to below 1 ~ 2 nN. All parameters were obtained from manufacturer. *R*a is the average roughness in analytical area, and *R*q means the root mean square roughness.

After scanning of cellular topographic images, various locations on a cell were selected to obtain the force-distance curves by the force-modulate mode AFM. All force-distance curve experiments were performed at the same loading rate. Twenty force-distance curves were acquired from each cell; five different cells should be detected in each group.

The AFM micro-cantilever free-end probe is indefinitely close to the cell; the probe which contacts the cell surface has shape change and separate from the cell so as to obtain the force-distance curve. Adhesion forces were induced by the interactions between the tip and cell membranes which could be extracted from the force curves using instrument’s software. Hertz model is usually adopted for the measurement of Young’s modulus. The calculation formula is as follows:

F=43ER12δ321−υ2

*F* is loading force; *E* is Young’s modulus; *R* is curvature radius of AFM tip; *δ* is the indentation, and *υ* is the Poisson ratio (usually 0.5 is adopted for the cell) [20,21].

### Laser confocal scanning microscopy and observation

ADS, 12DD, 21DD, and normal chondrocytes (NC) were washed with phosphate buffered solution (PBS) three times, fixed in 4% paraformaldehyde for 15 min at room temperature, then washed with PBS again and blocked with unimmunized goat serum for 10 min at 37°C before incubating with primary antibodies (rabbit anti-human integrin β1) for 20 min. After washing with PBS, the cells were incubated with rhodamine-conjugated rat anti-rabbit (1:100) secondary antibody (Biotium Inc., Hayward, CA, USA) at 37°C for 1 h to label integrin β1. Then the cells were identified by counterstaining with 4′,6-diamidino-2-phenylindole (DAPI) for 10 min in the dark. After washing with PBS, the labeled cells were observed using a laser confocal scanning microscopy (LCM 510 Meta Duo Scan, Carl Zeiss, Oberkochen, Germany).

### Flow cytometry

ADS, 12DD, 21DD, and NC were prepared for integrin β1 marker. A number of 1 × 10^6^ cells were incubated with PE-conjugated integrin β1 antibodies at 37°C for 1 h in the dark. Then the cells were centrifuged and washed in PBS three times. Finally, cells were acquired by use of a FACScan (Becton Dickinson, Franklin Lakes, NJ, USA) flow cytometer running its accompanying CellQuest software.

### Statistical analysis

All data were mean values ± standard deviation (SD). Statistical analysis was performed using one-way analysis of variance test (SPSS17.0), with *P* < 0.05 regarded as statistical significance.

## Results

### Detection of *SOX9*, *COL II*, *COL I*, and *Aggrecan* genes by real-time RT-PCR

**Figure 1:**
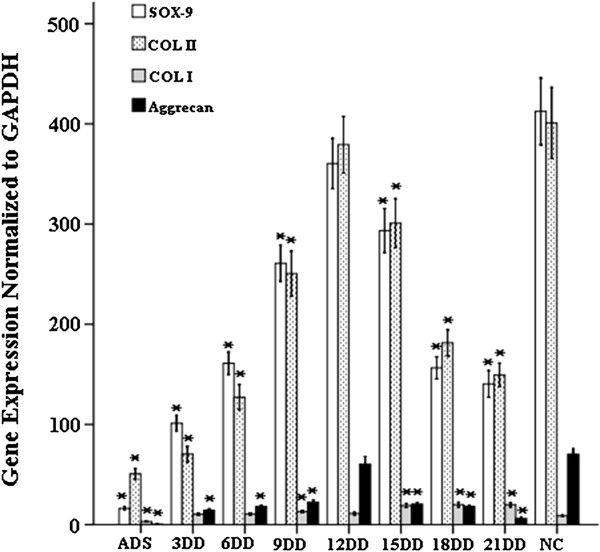
**Gene expression analysis during chondrogenesis of ADSCs.** ADSCs were cultured for up to 21 days. RNA extracts at day 0, 3, 6, 9, 12, 15, 18, and 21 were analyzed for gene expression of *SOX9*, *COL II*, *COL I*, and *Aggrecan* normalized to NC, respectively. Asterisk indicates *P* < 0.05 (vs. NC) as determined by one-way analysis of variance.

We used real-time RT-PCR to detect the expression of *SOX9*, *COL II*, *COL I*, and *Aggrecan* genes from the following nine groups: ADSCs group (ADS), normal chondrocytes group (NC), 3-day differentiation group (3DD), 6-day differentiation group (6DD), 9-day differentiation group (9DD), 12-day differentiation group (12DD), 15-day differentiation group (15DD), 18-day differentiation group (18DD), and 21-day differentiation group (21DD) (Figure 1). After addition of inducing medium, the expression of *COL II*, *SOX9*, and *Aggrecan* mRNA began to increase gradually, reaching a peak similar to that of NC at 12th day. At 18th day, expression of these genes dropped to the level of the 6th day. Change of *COL I* mRNA was clearly detected until in group 15DD. Its expression was sevenfold higher than in ADS and maintained at high levels through day 21. These results indicate that ADSCs after 12 days of differentiation express most of the chondrocytic gene markers, suggesting that they have differentiated into normal chondrocytes. After differentiating into mature chondroid cells, the expression of the markers was reduced gradually and over time dedifferentiation began.

### Atomic force microscopy analysis

#### Cell topography

**Figure 2:**
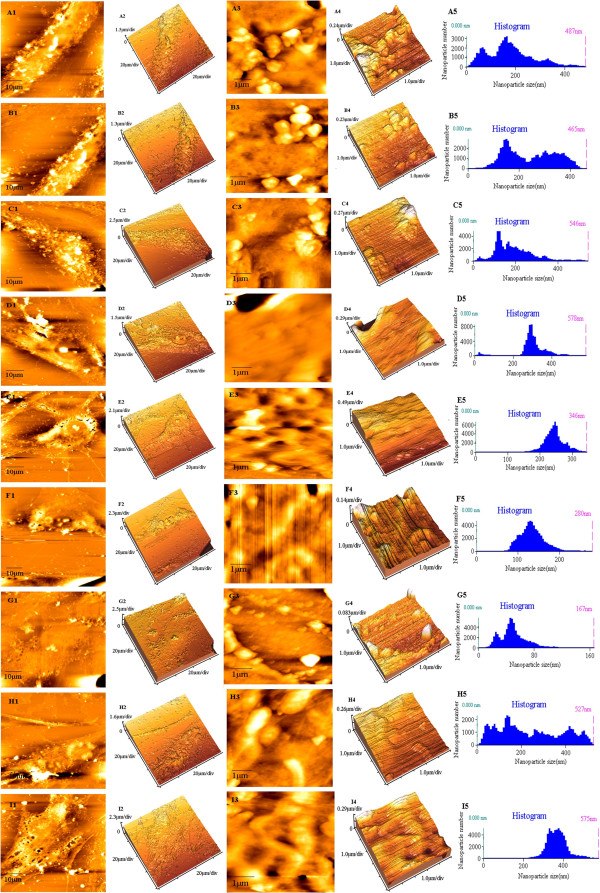
**AFM images of the nine groups.** AFM images of ADS (A1-A5), 3DD (B1-B5), 6DD (C1-C5), 9DD (D1-D5), 12DD (E1-E5), 15DD (F1-F5), 18DD (G1-G5), 21DD (H1-H5) and NC (I1-I5). (A1-I1) AFM images (scanning area 70 × 70 μm^2^); (A2-I2) 3D images; (A3-I3) nanostructural images (scanning area 5 × 5 μm^2^); (A4-I4) 3D images of nanostructure; (A5-I5) histograms of the particles size extracted from images A4-I4, respectively.

The topography and the three-dimensional morphology of cells could be observed through AFM. Both 12DD and NC both took the shape of an irregular triangle or polygon with a flat and extended nucleus (Figure 2, E1, E2, I1, and I2). It was difficult to distinguish 12DD and NC by appearance. ADS cells were an irregular, long spindle shape with one round and extruded nucleus (Figure 2, A1 and A2). Both 3DD and 6DD had an irregular spindle shape like ADS, but the synapse of the long axis had been distinctively shortened. Also, 21DD transformed into spindle shape with prominent structure, as shown in Figure 2, H1 and H2.

Further scanning for local within small scale was conducted (scanning area 5 × 5 μm^2^). Membrane surface particles were clustered in ADS (Figure 2, A3 and A4), and the particle sizes were generally between 50 and 250 nm (Figure 2, A5). Surface particles of 3DD and 6DD were between 100 and 400 nm (Figure 2, B5 and C5) and clustered, but they were sparse and distributed randomly (Figure 2, B3, B4, C3, and C4). In contrast, the surface of 9DD was flat and uniform. Particle numbers were reduced, but the size range was narrower, between 250 and 300 nm (Figure 2, D3, D4, and D5). Some shallow and uniform cavities were observed on 12DD (Figure 2, E3 and E4), and the particles were between 200 and 300 nm. NC had a similar porous arrangement, but cavities were deeper and more irregular with larger particle size, between 300 and 400 nm (Figure 2, I3 and I4). Porous structure disappeared in 15DD, 18DD, and 21DD. The particle size was reduced and they were distributed in a line in 15DD and 18DD (Figure 2, F3, F4, G3, and G4). In 21DD (Figure 2, H3, and H4), membrane surface particles returned to a clustered distribution, while the sizes varied from 20 to 450 nm.

Membrane surface ultrastructures were measured with IP2.1 analysis software and geometric parameter values were obtained (see Table 2). 12DD had the maximum *R*q and *R*a values of the differentiation groups, yet the values were significantly less than those of NC. There was no obvious diversity between the appearances of 12DD and NC by viewing the ultrastructure, but the difference might arise from the local protein trend and roughness analysis. These showed that though 12DD had differentiated into mature chondroid cells, the amount of cell surface protein could not reach that of normal chondrocytes. Also, although the protein trend was overall a porous arrangement, the cavities were shallower and the arrangement was more regular.

**Table 2:** Morphological and biomechanical parameters of differentiated cells detected by AFM

**Group**	**Surface average roughness (*R*a) (nm)**	**Root mean square roughness (*R*q) (nm)**	**Adhesive force (pN)**	**Young’s modulus (kPa)**
ADS	46.700 ± 4.495^b^	72.450 ± 7.246^b^	182.326 ± 18.229^a^	1.597 ± 0.110^b^
3DD	71.155 ± 7.096^a,b^	106.448 ± 12.070^a,b^	200.254 ± 17.138^a^	2.059 ± 0.179^a,b^
6DD	72.407 ± 7.621^a,b^	106.721 ± 13.489^a,b^	261.688 ± 19.416^a,b^	2.314 ± 0.207^a,b^
9DD	85.044 ± 7.170^a,b^	104.311 ± 11.333^a,b^	301.049 ± 22.776^a,b^	2.405 ± 0.213^a^
12DD	220.847 ± 21.308^a,b^	300.940 ± 29.248^a,b^	410.440 ± 28.638^a,b^	2.711 ± 0.236^a^
15DD	169.844 ± 16.589^a,b^	218.186 ± 17.884 ^a,b^	369.682 ± 26.958^a,b^	2.996 ± 0.233^a^
18DD	154.426 ± 12.985^a,b^	180.992 ± 18.232^a,b^	306.807 ± 23.506^a,b^	3.090 ± 0.234^a^
21DD	116.913 ± 12.361^a,b^	151.729 ± 13.340^a,b^	181.895 ± 18.648^b^	3.518 ± 0.381^a,b^
NC	303.205 ± 29.475^a^	362.011 ± 35.296^a^	639.197 ± 47.678^a^	2.742 ± 0.200^a^

^a^Compared with ADS, *P* < 0.05; ^b^Compared with NC, *P* < 0.05.

#### Cell mechanics

**Figure 3:**
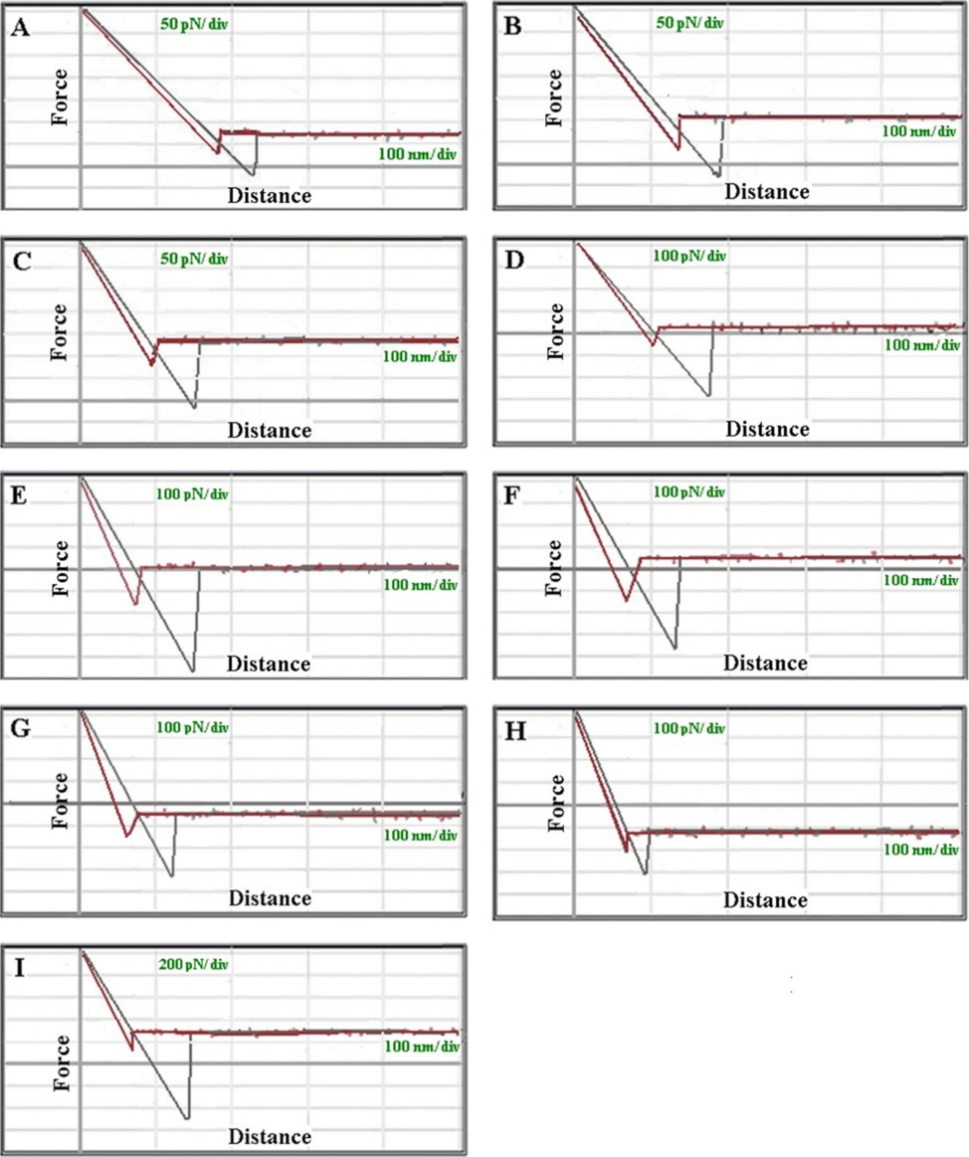
**Representative force-distance curves.** Longitudinal axis indicates force; horizontal axis indicates distance. (**A**) Force curve of ADS. (**B**) Force curve of 3DD. (**C**) Force curve of 6DD. (**D**) Force curve of 9DD. (**E**) Force curve of 12DD. (**F**) Force curve of 15DD. (**G**) Force curve of 18DD. (**H**) Force curve of 21DD. (**I**) Force curve of NC.

To analyze and compare the cells in each stage of differentiation, we assessed the mechanical property of the cell membrane by calculating the adhesion force and Young’s modulus from the force-distance curve. Adhesion force is the van der Waals force between the cell surface and the needle point, which is determined by measuring the retraction force of the needle point on the surface of cell membrane. This can be indicative of the content of membrane adhesion proteins. Force curves are schematically laid out for all nine samples in Figure 3. Our data shows that in the chondrogenic differentiation process, adhesion force gradually increases, reaching a maximum at 12DD (Table 2) before then decreasing gradually as differentiation continues. Changing the content of adhesion molecules could be responsible for the changes in adhesion force. Adhesion force reached the maximum at 12DD, indicating that adhesion proteins are involved in generating a mature chondroid cell, but this value still did not reach that of NC.

Young’s modulus is another valuable way to describe mechanical properties of cell membranes, and the value is calculated as described in the ‘Methods’ section. A larger Young’s modulus indicates that the cell was more difficult to deform, implying lower cell elasticity and greater stiffness. A comparison of the Young’s modulus of the samples is listed in Table 2. The value increased gradually during chondrogenic differentiation of ADSCs. Young’s modulus of 12DD was about twofold higher than ADS, equivalent to NC (*P* > 0.05). The maximum value of 3.518 ± 0.381 kPa was reached at 21DD.

### Laser confocal scanning microscopy and observation

**Figure 4:**
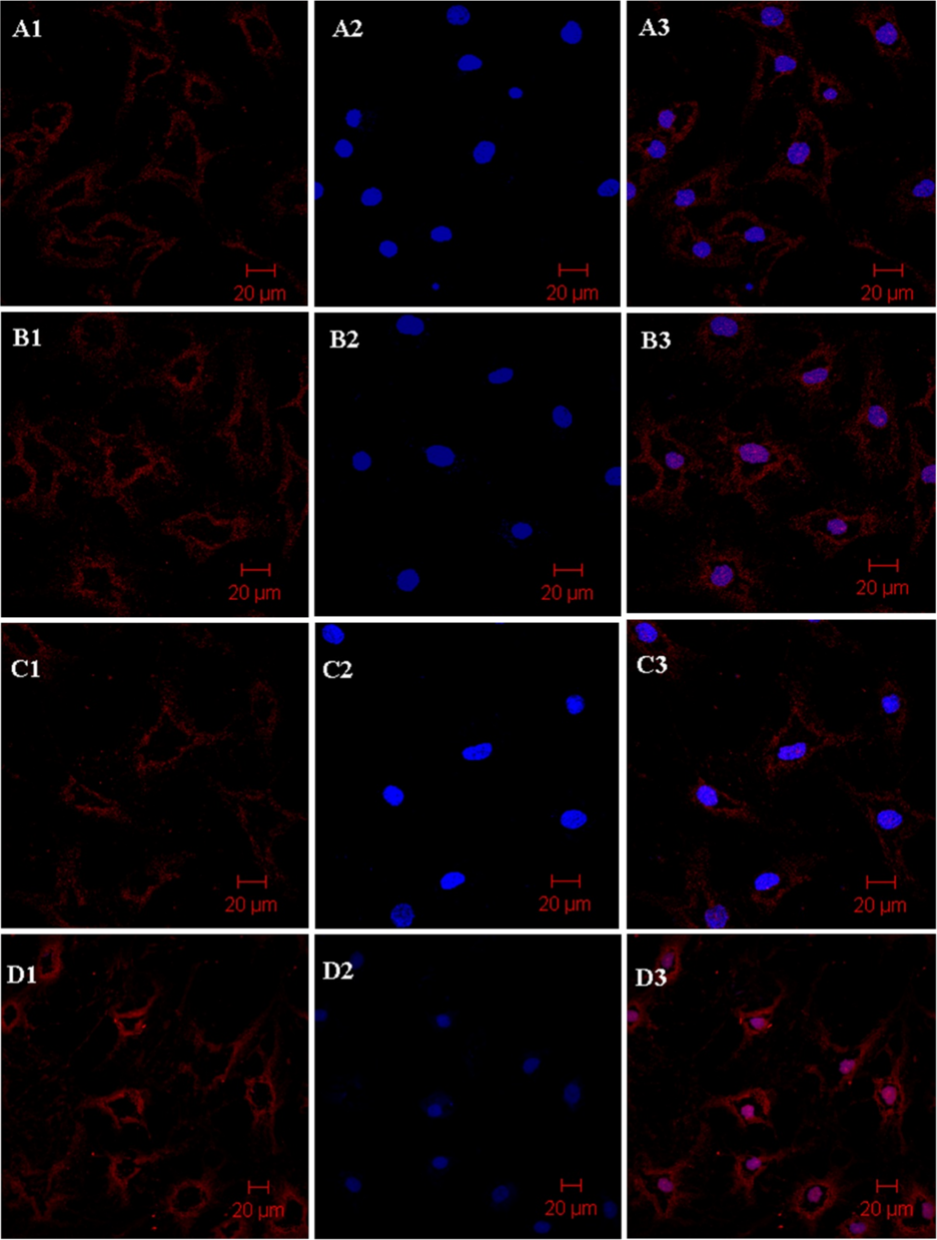
**LCSM analysis of ADS, 12DD, 21DD, and NC.** The cells were treated with antibodies to membrane surface protein integrin β1 (red channel) (**A1-D1**). Nuclei were counterstained with DAPI (blue channel) (**A2-D2**). For each channel, the top view is presented. An overlay shows the two channels of each cell (**A3-D3**). **A1-A3** showed ADS, **B1-B3** showed 12DD, **C1-C3** showed 21DD, and **D1-D3** showed NC.

We successfully conducted immunofluorescent staining of surface protein integrin β1 in four of the nine groups (ADS, 12DD, 21DD, NC). Integrin β1 was scattered across differentiated cell membranes but was found in local concentrations with a denser distribution on normal chondrocytes (Figure 4). We found that NC had the highest fluorescence intensity of integrin β1. With the chondrogenic differentiation of ADSCs, the fluorescence intensity of integrin β1 increased gradually until reaching a peak at 12DD. As in the other tests, this peak was still lower than NC. Also that of 21DD was clearly weaker than 12DD and NC.

### Integrin β1 content flow cytometry

Flow cytometry was used for the quantification of integrin β1 of four groups (ADS, 12DD, 21DD, and NC). Integrin β1 content of NC was the highest, up to 90.53%, followed by 12DD, which is 75.36%, and then 21DD and ADS had only 43.02% and 39.84%, respectively.

## Discussion

The RT-PCR results showed that ADSCs could be differentiated into chondroid cells expressing chondrocyte-specific markers such as *COL II*, *Aggrecan*, and *SOX9*. When differentiated to the 12th day, the expression of *COL II*, *Aggrecan*, and *SOX9* was close to that in normal chondrocytes, but subsequently fell. Therefore, through our PCR results, we inferred that ADSCs might be differentiated to mature chondroid cells at 12th day after induction, but after that their differentiated state is not maintained. Additionally, expression of the dedifferentiated marker gene *COL I* increased, behaving in an opposite manner to the differentiation markers. From this, we see that the extension of differentiation time does not improve the differentiation rate and indeed leads to dedifferentiation. Because no clear morphological markers of dedifferentiation are apparent under an inverted microscope, we employed other methods to observe the sequential morphological variation over the course of differentiation at nanometer scale. Because the cell membrane is not only a barrier between the intracellular environment and extracellular world but also a regulator of many important biological processes such as signal transduction, material transportation, and energy exchange, we looked for variation in the cell membrane structure accompanying with the change of cellular function; in this case, the level of differentiation.

AFM is a powerful tool for nanobiological studies [22], so we first used AFM to compare the ultrastructure of chondroid cells and NC and attempt to explain the relationship between cell dysfunction and its ultrastructure.

We obtained visual data of appearance and size, as well as dynamic changes of *R*a and *R*q on the nanometer scale using this method. In our experiment, we observed that ADSCs were irregular, long spindle shape with a round and extruded nucleus, but 12DD and NC were triangular or polygonal with flat and compact nuclei and endochylema. Both *R*a and *R*q in 12DD were close to those NC. Though there was no obvious morphological change with 21DD, we still obtained the change of *R*a data. The *R*a value of 21DD was reduced distinctly and membrane protein arrangement changed from regular porous arrangement to more of line and clusters. Taking the PCR data, we conclude that dedifferentiation after the 12th day is responsible for the ultrastructure changes. We hope the visual and quantitative data will be helpful in analyzing the differentiation process of ADSCs to mature chondroid cells and revealing a mechanism of cell destabilization in the late stage.

Obtaining of cell biomechanical data was another strength of AFM. Recent studies found that mechanical properties of a cell may be used as phenotypic biomarkers [23]. Therefore, we inferred that the functional change of cells caused by late stage dedifferentiation may also be observed through the cellular mechanics. To test this, we measured adhesion force and Young’s modulus across the whole differentiation process to further support the changes in function and cell surface ultrastructure.

Adhesion force mostly represents the number and distribution of cell surface adhesion molecules [24]. Our force-distance curve shows that during chondrogenic differentiation, adhesion force gradually increases to the maximum at the 12th day, but this value is slightly lower than that of NC, and then the value decreases as differentiation continues. Adhesion force corresponds to the change of *R*a. Our data demonstrate a trend of adhesion force that is in accordance with *R*a in the process of chondrogenic differentiation. Quantity and distribution of cell surface proteins directly affects *R*a data [25]. Surface particle numbers increased, causing the cell membrane to be uneven and rough thereby increasing *R*a. The higher adhesion force and *R*a value of 12th day are due to the increase of biomacromolecule particles on the mature chondroid cells, which interact more with the AFM needle. Likewise, as differentiation continued, there were fewer cell surface adhesion proteins, and the adhesion force and *R*a decreased. Thus, the dedifferentiation of chondroid cells was relative to the decrease of cell surface proteins.

Expression of adequate adhesion proteins is important for cells to attach in cartilage lacuna, which is necessary for stable synthesis and secretion of extracellular matrix (ECM) proteins. It is crucial for chondrocytes to remain differentiated to function properly. We chose integrin β1 as a representative adhesion protein for this experiment because it is widely expressed and is the main adhesion molecule in chondrocytes [26,27]. Then, we detected the distribution of integrin β1 through LCSM. We found integrin β1 on the cell membrane and the dynamic tracing of integrin β1 revealed a maximum fluorescence intensity of integrin β1 on the 12th day. In parallel, we used flow cytometry to test the quantity of integrin β1, and this supported the maximum at day 12, although the quantity did not reach that of NC. The qualitative and quantitative changes of integrin β1 in these groups correspond to *R*a and adhesion force results, so we conclude that dedifferentiation of chondroid cells may be directly related to loss or involution of integrin β1.

Acting as a bridge between ECM and the cytoskeleton, integrin not only transmits signals between the cell and the ECM but also regulates cytoskeletal arrangement and therefore cell rigidity [28,29]. We then wanted to test if the change of integrin β1 is accompanied with the change of cell rigidity, and we did so using AFM to measure cell Young’s modulus of each differentiation stage. We found that Young’s modulus increased gradually throughout the differentiation process. It came to the maximum at 21DD and was higher than NC in 15DD, 18DD, and 21DD. Young’s modulus of 12DD was similar to that of NC, having no statistically significant difference. Our data imply that 12DD had the most ideal stiffness and elasticity for chondrocytes.

The stiffness of cells is related to their physiological roles, and cartilage cells in particular require stiffness to bear and transmit a stress load. Reduction in elasticity would prevent the cartilage from buffering the vibrations from stress loads. We observed that the stiffness of chondroid cells increased continuously in the late stage differentiation, reducing cell deformability and perhaps causing cell degeneration. This is an important consideration in tissue engineering of cartilage as opposed to normal cartilage, because the continual increase in stiffness could negate the therapeutic effect of regenerative cartilage tissue. We speculate the improper rigidity of 21DD chondroid cells might be an objective manifestation and the intrinsic factor of degeneration.

## Conclusions

In general, the process of differentiating ADSCs into chondroid cells involves the synthetic process of integrin β1. We considered that chondroid cells mature when integrin β1 reaches its peak value. Degeneration and structural changes of integrin β1 distribution lead to dedifferentiation of chondroid cells. Therefore, integrin β1 may be responsible for the maturation and degeneration of chondrogenic differentiation of ADSCs.

## Competing interests

The authors declare that they have no competing interests.

## Authors' contributions

SML, QPS and SYS carried out the fabrication of samples and the AFM and LCSM measurements and drafted the manuscript. YP and HSL carried out the immunoassays. NL and HW performed the molecular genetic studies and participated in the sequence alignment. ZGZ and JYC initiated, planned, and controlled the research process. All authors read and approved the final manuscript.
